# Reduction of Aflatoxin B_1_ in Corn by Water-Assisted Microwaves Treatment and Its Effects on Corn Quality

**DOI:** 10.3390/toxins12090605

**Published:** 2020-09-20

**Authors:** Yaolei Zhang, Mengmeng Li, Yuanxiao Liu, Erqi Guan, Ke Bian

**Affiliations:** College of Food Science and Technology, Henan University of Technology, Zhengzhou 450001, China; zhangyaolei@stu.haut.edu.cn (Y.Z.); mengmeng.li@haut.edu.cn (M.L.); liuyuanxiao@stu.haut.edu.cn (Y.L.); guanerqi@haut.edu.cn (E.G.)

**Keywords:** aflatoxin B_1_, corn, water-assisted microwaves treatment, corn quality

## Abstract

Aflatoxin B_1_ (AFB_1_) is one of the most commonly found mycotoxin in corn, which is highly toxic, carcinogenic, teratogenic, and mutagenic for the health of humans and animals. In order to reduce the AFB_1_ in corn, corn kernels were processed with Water-assisted Microwaves Treatment (WMT) and the feasibility of WMT processing on AFB_1_ reduction and its effects on corn quality were analyzed. Increasing the treatment time and microwave power could increase the reduction of AFB_1_, and the maximum reduction rate could reach 58.6% and 56.8%, respectively. There was no significant correlation between the initial concentration of AFB_1_ and the reduction rate of AFB_1_. During WMT, the main toxigenic molds were sterilized completely, and the moisture content of corn climbed up and then declined to the initial level. WMT could obviously increase the fatty acid value and pasting temperature of corn and reduce the all paste viscosity of corn. However, it had little effect on the color of corn. The results indicated that WMT could reduce AFB_1_ effectively and avoid the vast appearance of heat-damaged kernels simultaneously. Undoubtedly, water played an important role in WMT. This result provides a new idea for the reduction of AFB_1_ by microwave.

## 1. Introduction

Corn is the primary crops in the world and used as a raw material of food, feed, and industrial products. However, corn is highly susceptible to the fungus *Aspergillus flavus*, thus resulting in aflatoxin formation [1]. Aflatoxins are secondary metabolites of *Aspergillus flavus*, and aflatoxin contamination in food and grains is a great concern all over the world. According to the United Food and Agriculture Organization of the United Nations (FAO), aflatoxin contamination in food and feed results in a huge worldwide economic loss each year. Aflatoxin B_1_(AFB_1_) is one type of aflatoxin, which has been shown to be an extremely potent liver carcinogen [2]. Due to the toxicity of AFB_1_, it has been classified as group 1 by the International Agency for Research on Cancer [3]. This makes the seeking of a proper method that will effectively reduce AFB_1_ in corn become a significant job. Over the last several decades, many physical, chemical, and biological methods for aflatoxins reduction have been reported. For instance, Conway [4] reported that the AFB_1_ in corn could be reduced 40–81% by roasting at 145–165 °C. Aquino [5] reported that AFB_1_ in corn could be partially and completely degradation by gamma rays at doses of 2 kGy and 10 kGy, respectively. Luo [6] reported that the AFB_1_ in corn with a moisture content of 20.37% could be reduced 72.4% after 40 min of treatment with 90 mg/L ozone. Sangare [7] reported that AFB_1_ could be degraded 82.6% by *Pseudomonas* strain N17-1 after incubation in Nutrient Broth (NB) medium at 37 °C for 72 h. Different methods have their advantages and disadvantages. For a successful detoxification method, the process must be economical, leave no harmful residues, and have little impaired to commodity quality [8].

Microwave has been widely used in the food industry because of its high efficiency of heating. Several studies have been reported that microwave heating was useful for the reduction of aflatoxin in peanut and corn [9,10]. In these researches, the degradation of AFB_1_ mainly depended on the high temperature produced by microwave heating, and the temperature of 130–150 °C or higher was required for achieving an effective reduction of AFB_1_. However, in practice, it had some major drawbacks when relying on high temperatures to reduce AFB_1_ in corn. For instance, the rapid heating of microwave usually significantly increased the crack rate and heat-damaged kernels of corn because of the volumetric heat generation, and possible textural damage due to difficulty to control the final product temperature [11]. Moreover, the large penetration depth of microwave often formed a higher temperature inside the grain kernel compared with the surface, which causes unnecessary heat damage to the inner tissue of maize. Because the AFB_1_ mainly distributed on the surface of the grain [12]. Besides this, it was easy to become popcorn because of the heating characteristics of the microwave for the full corn kernels with the approximately spherical shape, whereas AFB_1_ in corn was mostly distributed in broken kernels rather than full kernels. These greatly limited the use of microwaves to degrade AFB_1_ in corn.

At the same time, the relationship between degradation of AFB_1_ and water had been reported in many studies [13,14]. According to these reports, AFB_1_ was stable up to their melting points with dry heating and the thermal degradation temperature of AFB_1_ was up to 300 °C [15]. However, the presence of water molecules helped in opening the lactone ring in AFB_1_ to form a terminal carboxylic acid [16] and lowered the decomposition temperature of AFB_1_ [13]. Hu [17] reported that AFB_1_ standard substance in water could be degraded at 80 °C with microwave heating while a temperature above 200 °C was required for AFB_1_ degradation in edible oils [13]. Similar results were reported by Mobeen [18]. It might be a valuable research to used water as microwave heating mediums to reduce AFB_1_ in corn. In the literature, there were some researchers reported that the combination of water and microwaves had high potential as a postharvest disinfestation treatment [19]. However, the information on the combined effects of water and microwaves on AFB_1_ reduction of corn was lacking.

In the present study, the corn kernels containing AFB_1_ were submerged in water and then treated in a microwave oven. We named this method water-assisted microwave treatment (WMT). The effect of WMT on the reduction of AFB_1_-contaminated corn at different processing times, microwave power levels, and initial AFB_1_ contamination of corn were preliminary researched. The purposes of these studies were to evaluate the feasibility of WMT to reduce AFB_1_, study whether WMT could avoid producing heat-damaged kernels in corn, and analyze the role of water during the reduction of AFB_1_ by WMT. Meanwhile, the main quality changes of corn during the processing were also studied. This study could provide basic and valuable information for the reduction of AFB_1_ in corn.

## 2. Results

### 2.1. Reduction of AFB_1_ by WMT

#### 2.1.1. Effect of WMT Time on AFB_1_ Reduction

**Figure 1:**
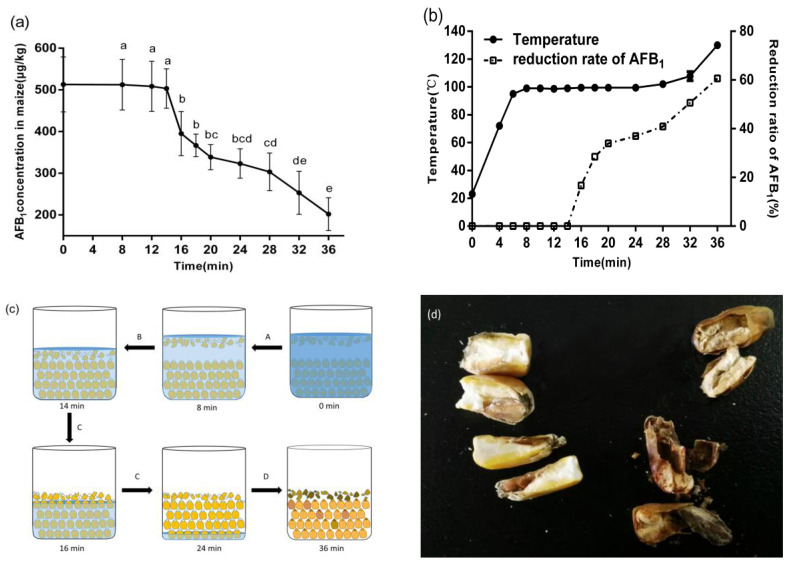
(**a**) Effect of processing time on the contents of AFB_1_ in corn kernels, (**b**) effect of processing time on the reduction rate of AFB_1_ and the temperature in corn kernels, (**c**) schematic diagram of water-assisted microwave treatment (WMT) process of corn, and (**d**) sectional drawing of corn kernel (36 min). Data are given as means of triplicate assays ± SD (standard deviation). Values with different letters on the same kind of bars are significantly different (*p* < 0.05). The bars are labeled by the letters a to e from the highest to the lowest.

In order to study the effect of WMT time on the reduction of AFB_1_, the other two factors were set as follows: microwave power, 500 W; AFB_1_ concentration of corn, 513.12 μg/kg. The effect of WMT time on AFB_1_ concentration in corn showed in Figure 1a. The significant decrease in AFB_1_ concentration occurred after WMT for 14 min. Then, with the extension of microwave treatment time, AFB_1_ concentration in corn decreased continuously. After cooking for 36 min, the AFB_1_ concentration of corn was reduced to a minimum (210.05 ± 39.45 μg/kg), and the reduction rate reached a maximum of 58.6% (Figure 1b).

According to the changes in temperature and the reduction rate of AFB_1_, the WMT process could be divided into four parts (Figure 1b,c). Stage A (0 min~8 min): water molecules absorbed microwave energy and resulting in the temperature of the whole cooking system continuously increased to the 100 °C. During this process, there was no reduction of AFB_1_ in corn. Stage B (8 min~14 min): the water began to boil violently at a constant temperature of 100 °C, and the reduction rate of AFB_1_ in corn still did not increase. Stage C (14 min~24 min): The temperature remained at 100 °C, but the reduction ratio of AFB_1_ in corn started to increase continuously. Stage D (24 min~36 min): the reduction ratio of AFB_1_ in corn continued to increase, and the temperature began to increase gradually and reached 140 °C finally. The whole cooking process can be described as in Figure 1c and the real figure of sectional drawing of corn kernel after WMT 36 min is shown in Figure 1d.

#### 2.1.2. Effect of Microwave Power on AFB_1_ Reduction

**Figure 2:**
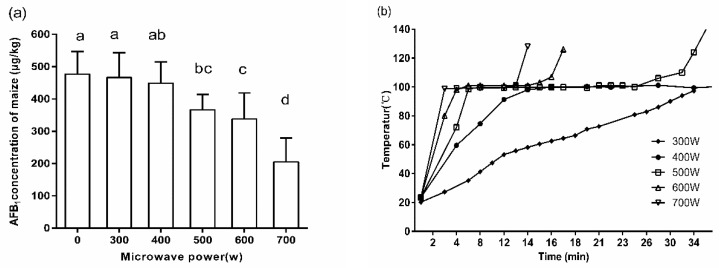
(**a**) Effect of different microwave power on the concentration of AFB_1_ in corn kernels, and (**b**) temperature variation of corn under different microwave power setting. Data are given as means of triplicate assays ± SD (standard deviation). Values with different letters on the same kind of bars are significantly different (*p* < 0.05). The bars are labeled by the letters a to d from the highest to the lowest.

In order to study the effect of microwave power on the reduction of AFB_1_, the other two factors were set as follows: microwave cooking time, 16 min; AFB_1_ concentration of corn, 484.12 ± 85.98 μg/kg. When the microwave power was lower than 400 W, AFB_1_ concentration in corn did not decrease significantly (Figure 2a). However, when the power continued to increase to 500 W, 600 W, and 700 W, the content of AFB_1_ in corn significantly decreased by 23.1%, 29.0%, and 56.8%, compared to the control team, respectively.

The temperature changed in the process of WMT with different microwave power could be described in Figure 2b. Increasing the microwave power could significantly shorten the heating time of corn from 20 °C to 100 °C. The time needed for this process at 300 W, 400 W, 500 W, 600 W, and 700 W was 34 min, 14 min, 8 min, 4 min, and 3 min, respectively. In addition, the temperature of corn cooked under different microwave power for 16 min was 60 °C, 100 °C, 110 °C, and 146 °C, respectively.

#### 2.1.3. Effect of Initial AFB_1_ Concentration on AFB_1_ Reduction

**Figure 3:**
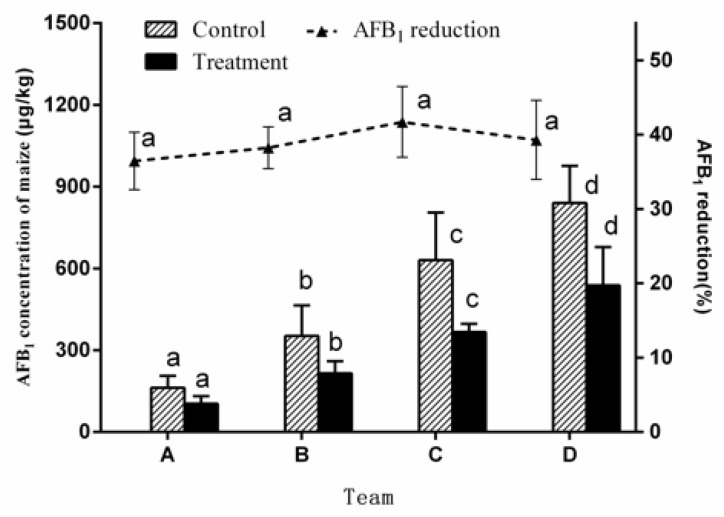
Effect of initial AFB_1_ concentration on the AFB_1_ reduction of corn kernels by WMT. Data are given as means of triplicate assays ± SD (standard deviation). Values with different letters on the same kind of bars are significantly different (*p* < 0.05). The bars are labeled by the letters a to d from the lowest to the highest.

In order to study the effect of initial AFB_1_ concentration on the reduction of AFB_1_, the other two factors were set as follows: microwave power, 500 W; microwave time, 20 min. The effect of initial AFB_1_ concentration on aflatoxin reduction could be observed in Figure 3. Team A, B, C, and D meant the corn inoculated with *Aspergillus flavus* was cultured at 30 °C for 4, 6, 8, and 10 days and their AFB_1_ concentration was 182.25 g/kg, 352.19 g/kg, 630.78 g/kg, and 840.89 g/kg, respectively. After 20 min of WMT, the amount of AFB_1_ reduced increased with the initial aflatoxins concentration in the corn. However, the reduction rate of AFB_1_ did not increase with the change of concentration of AFB_1_, and the aflatoxin reduction was 36.4%, 38.2%, 41.7%, and 39.2%, respectively. No correlation was found between the percent reduction and the initial concentration of AFB_1_ in corn by ANOVA.

### 2.2. The Variation of Corn Quality during WMT

#### 2.2.1. Moisture Content and Color

The changes in moisture and color of corn during WMT are shown in Table 1. During the whole WMT process, the moisture content of corn kernels varied with the trend of increased at first and then decreased with the increasing of processing time. In the early stage of cooking (0~12 min), the moisture content of corn increased the speed of 1.92% per minute. After that (12 min~20 min), the increased speed of water content gradually slowed down and reached the peak value of 37.63% at 20 min. From 20 min to 36 min, the water content of corn began to decrease and the decreasing rate increased. At the end of 36 min, the moisture content of corn decreased to 12.13%, which was closed to the initial moisture of corn. Besides this, the distribution of water between corn kernels was uneven during WMT. The damaged corn kernels and corn embryos were moist and soft because of absorbing large amounts of water, while intact corn kernels and their endosperm tissues were relatively hard.

During the whole WMT Period (0–36 min), the L* value showed a decreasing trend and the a* value showed an increasing trend, while b* did not change significantly. The rapid decrease of the L* value and the rapid increase of the a* value occurred almost simultaneously after 28 min. The color change of corn during the whole cooking process could be seen more directly, as shown in Table 2. The heat-damaged corn kernels obviously appeared after 36 min. Moreover, the color of different corn kernels was quite different at that moment. Most corn kernels still kept their original yellow color, and only part of the corn kernels turned dark and burnt.

#### 2.2.2. Mold Number and Free Fatty Acid Value

**Figure 4:**
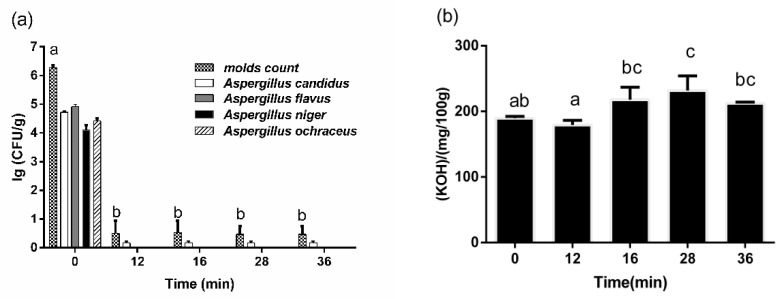
(**a**) Effect of processing time on the number of mold in corn, and (**b**) effect of processing time on fatty acid value in corn. Data are given as means of triplicate assays ± SD (standard deviation). Values with different letters on the same kind of bars are significantly different (*p* < 0.05). The bars are labeled by the letters a to b from the highest to the lowest in Figure 4a and a to c from the lowest to the highest in Figure 4b.

The changes in mold number and free fatty acid value during WMT were described in Figure 4. Before WMT, the total number of mold colonies in corn was more than 10^6^ CFU/g, and the number of major molds such as *Aspergillus candidus*, *Aspergillus flavus*, *Aspergillus niger*, and *Aspergillus ochraceus* had exceeded 10^4^ CFU/g. After WMT for 12 min, the total number of mold was less than 10 CFU/g and most of the mold could not be detected except a few *Aspergillus candidus*. The fatty acid value was an important index to measure the quality of corn. It increased from 189.59 (KOH)/(mg/100 g) to 231.85 ± 22.33 (KOH)/(mg/100 g) in the whole process and mainly happened at 16 min.

#### 2.2.3. Pasting Properties of Corn

The pasting properties of corn powder were very important to the application of corn. A significant difference in the pasting properties of corn by the processing time was observed (Table 3). In other words, the corn powder showed a continuous decrease in viscosity with the increase of processing time. Peak viscosity (PV) of corn powder ranged from 1690 to 245 m Pa.s. The same tendency occurred for trough viscosity (TV), breakdown viscosity (BV) (measure of the cooked starch to disintegration), final viscosity (FV) (indicating the ability of the starch to form a viscous paste), and setback viscosity (SV) (measure of synaeresis of starch upon cooling of the cooked starch pastes). Pasting temperature (PT) (temperature at the onset of rise in viscosity) for corn powder ranged from 84.5 °C to 94.8 °C, the lowest shown in 0 min and the highest in 28 min. The same trend happened in the Pasting time (PT).

## 3. Discussion

In our study, WMT was effective in reducing AFB_1_ in corn. Different from the reduction of AFB_1_ by previous microwave roasting, the WMT process involves a lot of water, which led to the unique characteristics of the degradation of AFB_1_. The reduction of AFB_1_ in corn by WMT could be divided into four stages (A, B, C, and D) according to the changes in temperature, moisture content, and AFB_1_ content with processing time. The characteristics of each stage are described in Section 2.1.1.

Stage A could be considered as a process of temperature rising by WMT. There was no reduction of AFB_1_ in corn because of the lower temperature of the whole system. Stage B had the same temperature with stage C, but there was no reduction of AFB_1_ in stage B. One possible reason was that the corn was immersed in water completely at this stage. The water acted as a barrier, so most of the microwave energy was absorbed by the water rather than by the corn. For instance, the water was usually used as a barrier to prevent the leakage of microwave energy from instruments to avoid harm to the human body in the microwave industry [20]. On the other hand, it suggested that short-time cooking with excess water could not effectively reduce AFB_1_ in corn. The same result was reported in rice [21]. It also means that microwave extraction could not remove AFB_1_ from corn kernels. Stage C was the most meaningful stage of AFB_1_ reduction during the WMT. It was that the kernels began to emerge from the water and exposed to microwave radiation directly due to the evaporation of water. However, the rich moisture of corn (30~40%) absorbed at the stages A and B, ensuring that the temperature of the corn was maintained at 100 °C rather than further increased at microwave heating processing. By the end of stage C, the degradation rate of AFB_1_ reached 38%. In Mann’s study [15], 34% reduction of aflatoxins in a peanut meal (30% moisture content) was found when it was heated at 100 °C for 2 h. However, a similar AFB_1_ reduction rate by WMT (100 °C) only needed 10 min in our study. It reflected the advantages of microwave in the thermal degradation of AFB_1_, because microwave heating could speed up the chemical reaction process and shorten the reaction time compared with conventional heating [22,23]. After entering the stage D (24 min~36 min), the water in the beaker had evaporated basically, and the moisture content of corn continually decreased to 12.13%, because of the microwave dehydration. At the end of this stage, the process of corn exposed to the microwave field appears to be similar to that of microwave roasting. With the increase in temperature, the reduction rate of AFB_1_ increased [10].

The effect of microwave power on the AFB_1_ reduction rate was studied. Microwave power did not affect the trend of the whole cooking process, but affected the duration of the whole process. For instance, the whole cooking process only lasted for 14 min at high microwave power (700 W), while it would take 60 min at low power (200 W). Compared with low power treatment, higher microwave power could make the cooking process enter stage C and D earlier and achieve more reduction of AFB_1_ at the same time. However, higher microwave power would shorten the stage C, which was the most important stage in the reduction of AFB_1_. Moreover, the higher power would make the dehydration rate of corn too fast, thus significantly increasing the crack rate of corn [24]. Therefore, the matching of different cooking stages with appropriate power may be the next stage of the research objective.

The initial concentration of AFB1 in corn had little effect on the AFB_1_ reduction rate. These results were in agreement with the result of Pluyer [9], who used naturally contaminated peanuts to study aflatoxin destruction by microwave roasting, and their results also showed no correlation between aflatoxin initial concentration and the percentage of destruction. However, there were also some different reports. For instance, Lee [25] reported that the more the initial aflatoxin level increases, the more the aflatoxin reduction increases. Arzandeh [26] reported that an increase in initial aflatoxin concentration causes an increase in aflatoxin reduction, whereas whenever the initial aflatoxin concentration exceeded 200 ng/g, there was a decrease in aflatoxin reduction. The reason for these results might be related to the different matrices and initial AFB_1_ concentration used by these researchers and the current study. The different analytical techniques used to quantify the aflatoxins, the different extraction methods and the different moisture contents of the samples could partially account for the different results [9].

In addition, it was important that water played an important role in the reduction of AFB_1_ during WMT. The participation of water had the following three unique advantages: (1) With the help of water, the corn was automatically graded. The moth-eaten and mechanical damage kernels would be floated above the water surface because of its relatively low density (Figure 1c). Previous studies showed that the content of AFB_1_ in this damaged kernels was more than 80% of the total AFB_1_ in corn [27]. Those highly toxic kernels, which float above the water surface, would be exposed to microwave radiation firstly. This might help to explain the rapid increase in AFB_1_ reduction at the beginning of stage C. (2) A large amount of moisture in corn ensured that the temperature of corn under microwave irradiation does not exceed 120 °C during the whole cooking process, except at the end of stage D. Therefore, the color of the corn did not darken due to high temperature, and effectively inhibited the appearance of heat-damaged kernels and burnt smell. That was an important advantage of WMT in reducing AFB_1_ compared with microwave roasting. (3) Due to the strong absorption of microwaves by water molecules, the water molecules in corn were like a guide in the microwave electromagnetic field, which direct microwave energy to attack some parts of the corn with high AFB_1_ concentration. It was because the areas of high AFB_1_ concentration (embryo tissue and broken grain) in corn kernel were also the areas with high water content. There were three reasons for this phenomenon: Firstly, due to the relatively short cooking time of corn (stage A, B, and C), the tissues of the corn kernel did not fully saturate with water. Secondly, the corn embryo was rich in protein, which has stronger hydrophilicity than starch. Thirdly, the specific surface area increased after the kernels were broken, and the inner structure was exposed to the outside at the same time, which was also favorable for water entering into the kernel. Because the microwaves were strongly absorbed by water, these areas would receive more microwave energy. The sectional drawing of corn kernels at the end of WMT (36 min) was a strong proof, i.e., the degree of microwave heating among corn kernels was different (Figure 1d). The full kernels still had the loose structure of the Chalky endosperm and the white color of the starch, suggesting that the microwave energy did not penetrate too much into the kernel. Otherwise, the interior of the kernel would be heated more because of the greater penetration depth of the microwave. On the other hand, corn embryos and irregularly shaped damaged seeds were blackened and charring obviously. It proved that these areas absorbed most of the microwave energy. These features reflected the potential advantages of WMT in reducing AFB_1_ in corn.

Besides that, the microwave could kill microorganisms and dehydrate corn efficiently, which brought another advantage of WMT. For instance, the moisture content of corn decreased continuously in the later period of WMT, and finally decreased to less than safe moisture condition (14.0%). Almost all the molds were killed at the early WMT because of the higher ability of microwave sterilization, including AFB_1_-producing *Aspergillus flavus* [28]. These two results can effectively avoid the risk of mold growth and mycotoxin production again after being processed, which is of great significance for safe storage.

At last, the changes in free fatty acid value and pasting properties of corn were researched during WMT. Higher temperatures usually caused the increase of the free fatty acid value of corn in the processing of heat treatment [29]. However, due to the fungal infection, the corn samples had severe rancidity and the free fatty acid content had been up to 200 (KOH)/(mg/100 g). This might limit further increases in free fatty acid values. As for pasting properties of corn powder, all the paste properties were reduced and the pasting temperature was elevated for starch after WMT, which indicated that the WMT could change the pasting properties of corn significantly. The result might be related to an alteration of the starch granules, because microwave irradiation could reduce the crystallinity, solubility, and swelling characteristics of corn starch [30]. Another explanation might be the production of large amounts of resistant starch after WMT. This is because thermal treatment and high moisture content and cooling processes were the important factors that affect the production of resistant starch [31]. The same results appeared in David’s experiments [32]. Therefore, it might be an important application to use the corn treated by WMT to produce resistant starch. In addition, microwave irradiation as a method for starch modification had been reported in many researches [33]. It might be meaningful to take into account both the reduction of AFB_1_ in corn and the modification of corn starch by WMT. That is because it helps to expand the use of corn contaminated by AFB_1_ and reduce the economic loss caused by corn contaminated by AFB_1_.

## 4. Conclusions

WMT could reduce AFB_1_ effectively and avoid the vast appearance of heat-damaged kernels simultaneously. Water played an important role in WMT. Firstly, water could automatically classify the corn kernels by relative density, so the damaged corn kernels with high toxin content would be exposed to microwave radiation firstly, which was beneficial to the reduction of AFB_1_. Secondly, the non-uniform distribution of water content among corn kernels was favorable for the microwave to attack the parts with high AFB_1_ contamination. Finally, the presence of water could keep the corn at a lower temperature during AFB_1_ reduction, which helped to avoid producing the heat-damage kernels of corn. Increasing microwave power could shorten the cooking process and reduce AFB_1_ in a short time. There was no significant correlation between the initial concentration of AFB_1_ and the reduction rate of AFB_1_. After WMT, the main toxigenic molds were sterilized completely, and the moisture content of corn was kept relatively low, which was beneficial to avoid the corn mildew again. Moreover, WMT could obviously increase the fatty acid value of corn and affect the pasting properties of corn. This result provides a new idea for the reduction of AFB_1_ by microwave.

## 5. Materials and Methods

### 5.1. Chemicals and Reagents

Aflatoxin B_1_ was purchased from J&K Chemical Ltd. (Shanghai, China). The purity of these materials was 99.5%. AFB_1_ standard was dissolved in Acetonitrile and diluted to a concentration of 1 mg·L^−1^, and the AFB_1_ standard solution was stored at −20 °C in a freezer before conducting the experiment. acetonitrile and methanol were purchased from Thermo Fisher Scientific (Shanghai, China). Corn samples (local variety) were brought from the Local farmers (Zhengzhou city, Henan Province, China).

For the UHPLC-MS analysis, ultrapure water (18.2 MΩ cm^−1^ resistivity) was obtained from a Milli-Q SP Reagent Water system (Millipore, Bedford, MA, USA) and prefiltered through a 0.2-μm membrane.

### 5.2. Aspergillus Flavus

*Aspergillus flavus* was isolated from mildew corn, which was obtained from farmland of Zhengzhou city, Henan Province, China. *A. flavus* was grown on potato-dextrose-agar (PDA) slant for 7 days at 28 °C. Spores were harvest by adding sterilized water, filtering through eight layers of sterilized cheesecloth, washing several times with sterilized distilled water, and resuspending in sterilized distilled water [10].

### 5.3. Infection of Corn

The spores were scraped by adding sterile distilled water to the surface of the PDA slant and the resulting spore suspension was added to corn samples. Then, the corn was incubated at 30 °C with the moisture content of 19% for 4, 6, 8, and 10 days to allow *Aspergillus flavus* growth and AFB_1_ production [10]. AFB_1_ concentration produced by *Aspergillus flavus* increased gradually with the extension of storage time. Finally, the corn was transferred to plastic bags and stored at −20 °C.

### 5.4. Water-Assisted Microwave Treatment

**Figure 5:**
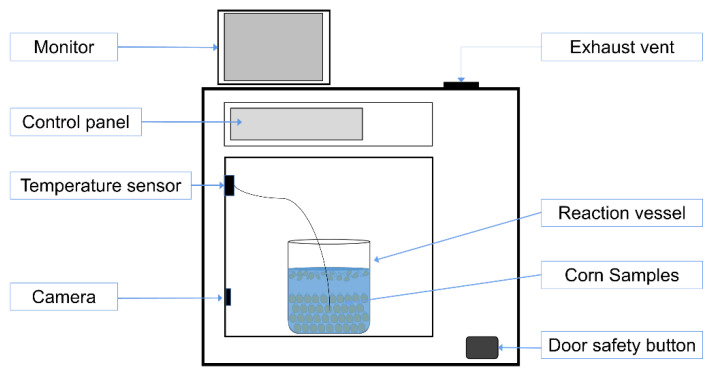
Schematic view of the microwave cooking device.

The 200 g of corn kernels (containing AFB_1_) and 200 mL of distilled water (electrical conductance ≤ 10 μS/cm) were added into a glass beaker (1000 mL) successively. Most of the corn kernels were completely submerged by water, and the water surface was 1 cm approximately above the top of the corn pile. A small part of broken corn kernels floated on the water. Then, they were placed in a type XH-8000 Plus multi-function microwave synthesizer microwave oven (Figure 5). The microwave oven was manufactured by Beijing Xianghu Science and Technology Development Co., Ltd. (Beijing, China). During the microwave working, the temperature was detected with a fiber optic sensor inserted in the vessel, and shown on the control panel. The status of the reactor was observed using the monitor with a connected camera inside the oven.

The corn samples were treated by WMT for various periods (8, 12, 14, 16, 20, 24, 28, 32, and 36 min). The microwave power was changed from 300 W to 700 W (300 W, 400 W, 500 W, 600 W, and 700 W). When the processing procedure was finished, the glass beaker was moved out of the microwave oven and then the corn kernels were removed. The water on the surface of corn kernels was absorbed with absorbent paper. After cooling at room temperature, the processed corn kernels were moved into airtight bags and stored at room temperature for no more than 24 h before grinding. Then, the processed corn kernels were grinded to determine the AFB_1_ concentration and corn quality. If corn kernels had high moisture and could not be ground, they needed to be air-dried first.

### 5.5. UHPLC-MS Analysis

A portion of 5 g of corn powder was weighed in an Erlenmeyer flask. AFB_1_ was extracted with 20 mL of a mixture of acetonitrile/water/glacial acetic acid (79:20:1, *v*/*v*/*v*). The slurry solution was shaken on a rotary shaker for 20 min at 100 rpm and then centrifuged at 4000 rpm for 20 min (25 °C). A volume of 1 mL of the supernatant obtained from previous centrifugation was diluted in 1 mL of Milli-Q water and then centrifuged at 12,000 rpm for 15 min (4 °C).

The supernatant of the extracts was then transferred to an Eppendorf tube (2.0 mL) for UHPLC-MS (mass spectrometry) analysis. UHPLC-MS data on detection of AFB_1_ was obtained using a Q-Orbitrap instrument system (Thermo Fisher Scientific, Waltham, MA, USA) equipped with a binary solvent delivery system and an autosampler. The pump used in our UHPLC was a bivariate gradient pump. Chromatography was performed using a 2.1 × 100 mm, 1.7 µm particle C18 column (Thermo Fisher). The injection volume was 5 µL and the flow rate was 300 µL/min. The mobile stage was a gradient prepared from the water with 0.1% formic acid and 5 mol/L ammonium acetate (component A) and methanol (component B). The gradient elution started with 25% B for 1 min, then B was increased linearly to 95% in 1 min, and kept isocratic for 2 min. The proportion of B was then decreased back to 25% and kept isocratic for 2 min. The total run time was 6 min. The MS was run with positive electrospray ionization (ESI), and the data was collected over the range of 150–600 *m*/*z*.

### 5.6. Color and Moisture Content Analysis

The CIELAB method was used to analyze the corn color after WMT treatment. Corn samples were ground so that more than 90% of the corn power could pass through a 0.25 mm aperture sieve. Then, the on-sieve and under-sieve were mixed evenly for color determination. The color reflectance spectrometry measurements of lightness (L*), redness (a*), and yellowness (b*) values were determined at ambient temperature using a Minolta CR410 chroma meter (Konica Minolta Sensing, Inc., Osaka, Japan). The L* value represents lightness, and the +a* and −a* values represent redness and greenness, respectively. The +b* and −b* values represent yellowness and blueness, respectively [34]. Three repeat measurements were taken for each corn sample, and the mean values were calculated and recorded.

The moisture content of corn was tested according to the AACC (American Association of Cereal Chemists) Method 44–15 A. Corn kernels were cooled to room temperature after WMT processing and the water on the surface of corn kernels was absorbed with absorbent paper. These corn samples were placed in a glass dish (15 cm in diameter) and their initial weight (m_1_) was recorded. Then, they were dried for 12–24 h at 45°C in an oven, so that the moisture content of the corn kernels was less than 10%. We weighed these corn kernels after they cooled to room temperature (m_2_). These corn kernels were ground with a laboratory mill (equipped with a 20-mesh screen) and we transferred immediately a 4- to 5-g portion to each of three tared moisture dishes; We covered and weighed dishes at once and subtracted tare weights and recorded weight of the sample (m_3_). Then, we uncovered the dishes and placed them on the shelf of an oven. Oven (mechanical-convection) temperature was maintained at 105 °C (±1 °C) for 3 or 6 h. The dishes were removed from the oven and covered rapidly (using rubber finger insulators), and then they were transferred to a desiccator as quickly as possible. The corn powder in dishes (subtract the weight of each dish from the total weight) were weighed after they cooled to room temperature (m_4_). Replicate determinations were checked within 0.2% moisture. The calculation method of moisture content was performed using Equation (1):

(1) $$\%\;\mathrm{Moisture}\;=\;\left[\frac{\left(m_{3}-m_{4}\right)\frac{m_{2}}{m_{3}}+\left(m_{1}-m_{2}\right)}{m_{1}}\right]\times 100\%$$

### 5.7. Microbiological Analysis of Samples

The identification of mold counts and microfloral species from corn samples were conducted according to the following method [35]: 25 g of each corn sample was withdrawn and added to 225 mL of sterile distilled water in a 1-L flask bottle. Then, they were shaken for 30 min on a flatbed shaker at 300 excursions per minute to completely dissolve the microorganism in the corn samples. The sample solution was diluted 10^2^–10^6^ times, and 1 mL of each diluted solution was plated on modified Czapek’s medium (3% sucrose, 2% agar powder, 0.05% KCl, 0.05%, MgSO_4_ × 7H_2_O, 0.1% K_2_HPO_4_, 0.3% NaNO_3_, and 6% NaCl) and incubated at 28 °C for 7 days. The mold count was calculated at day 5 and the microfloral species were identified using a microscope at day 7.

### 5.8. Free Fatty Acid Analysis of Samples

The determination of free fatty acid of corn samples was conducted according to the following method [36]. A test portion was extracted by petroleum ether for 10 min, filtrated, and the filtrate was added into 50% ethanol solution; then, it was titrated with a standard volumetric sodium hydroxide solution using phenolphthalein as indicator until the end-point was indicated by a sharp change of color from neutral to pink.

### 5.9. Analysis of Pasting Properties

Microwaved corn starch pasting properties were analyzed using a Rapid Visco Analyser (RVA-4, Foss North America, Eden Prairie, MN, USA), using a method previously used for starch suspensions [37]. Viscosity profiles of starches from corn were recorded using starch suspensions (6%, *w*/*w*; 28 g total weight). A programmed heating and cooling cycle were used, where the samples were held at 50 °C for 1 min, heated to 95 °C at 6 °C/min, held at 95 °C for 2.7 min, before cooling from 95 °C to 50 °C at 6 °C/min and holding at 50 °C for 2 min. Parameters recorded were pasting temperature, peak viscosity, trough viscosity (minimum viscosity at 95 °C), final viscosity (viscosity at 50 °C), breakdown viscosity (peak-trough viscosity), and setback viscosity (final-trough viscosity). All measurements were replicated thrice.

### 5.10. Statistical Analysis

All experiments were independently performed a minimum of three times and data was expressed as mean ± standard deviation (SD). Statistics were analyzed with SPSS 16.0 (SPSS Institute, Chicago, IL, USA). The statistical significance of the data was analyzed using Duncan’s multiple range post hoc tests (*p* < 0.05).

## Figures and Tables

**Table 1:** The moisture content and color analysis of corn by WMT.

Processing Time (min)	Moisture Content (%)	Rate of Moisture Increase (%/min)	L*	a*	b*
0	13.5	0	82.40 ± 0.06 ^g^	−2.30 ± 0.01 ^a^	21.21 ± 0.26 ^a^
12	32.77	1.92	81.84 ± 0.21 ^f^	−2.21 ± 0.02 ^b^	21.71 ± 0.71 ^ab^
16	36.43	0.61	81.63 ± 0.05 ^f^	−2.20 ± 0.05 ^b^	22.03 ± 0.05 ^bc^
20	37.63	0.24	81.26 ± 0.15 ^e^	−2.04 ± 0.03 ^c^	22.60 ± 0.35 ^c^
24	35.36	−0.75	80.83 ± 0.08 ^d^	−2.02 ± 0.04 ^c^	21.68 ± 0.53 ^ab^
28	31.93	−0.85	80.50 ± 0.17 ^c^	−1.82 ± 0.04 ^d^	22.10 ± 0.04 ^bc^
32	22.85	−1.82	75.83 ± 0.17 ^b^	−0.90 ± 0.05 ^e^	21.55 ± 0.32 ^ab^
36	12.13	−2.68	73.67 ± 0.12 ^a^	0.83 ± 0.03 ^f^	22.25 ± 0.36 ^bc^

Data are given as means of triplicate assays ± SD (standard deviation). Values with different letters in the column are significantly different (*p* < 0.05). The values are labeled by the letters a to g from the lowest to the highest. The L* value represents lightness, and the +a* and −a* values represent redness and greenness, respectively. The b* values represent yellowness.

**Table 2:** The color change of corn during the WMT process.

**Processing** **Time (min**)	**0**	**12**	**16**	**20**
	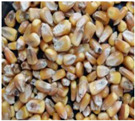	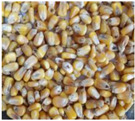	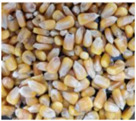	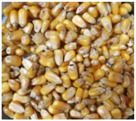
**Processing** **Time (min)**	**24**	**28**	**32**	**36**
	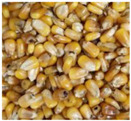	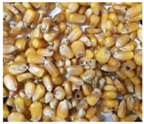	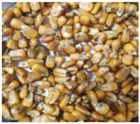	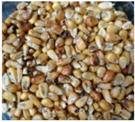

**Table 3:** Pasting properties of corn starches from different processing time.

Processing Time (min)	PV (cP)	TV (cP)	BV (cP)	FV (cP)	SV (cP)	P_Time_ (min)	P_Temp_ (°C)
0	1690.0 ± 225.8 ^a^	1425.3 ± 199.7 ^a^	264.6 ± 34.5 ^a^	3783.6 ± 489.9 ^a^	2358.3 ± 299.9 ^a^	5.7 ± 0.1 ^b^	84.5 ± 4.8 ^b^
12	1013.0 ± 113.8 ^b^	920.3 ± 138.5 ^b^	92.6 ± 31.6 ^b^	2135.0 ± 254.5 ^b^	1214.6 ± 124.2 ^b^	6.8 ± 0.2 ^a^	89.4 ± 3.8 ^ab^
16	814.0 ± 61.5 ^b^	720.3 ± 49.9 ^b^	93.6 ± 11.6 ^b^	1653.3 ± 100.8 ^c^	933.0 ± 51.5 ^c^	7.0 ± 0.1 ^a^	92.8 ± 0.1 ^a^
28	318.0 ± 22.5 ^c^	304.6 ± 21.1 ^c^	63.3 ± 13.6 ^bc^	779.6 ± 42.0 ^d^	475.0 ± 20.9 ^d^	6.9 ± 0.0 ^a^	94.8 ± 0.2 ^a^
36	245.0 ± 52.0 ^c^	217.3 ± 51.4 ^c^	27.6 ± 1.1 ^c^	497.0 ± 109.2 ^d^	279.6 ± 57.8 ^d^	7.0 ± 0.1 ^a^	-

PV, peak viscosity; TV, trough viscosity; BV, breakdown viscosity; FV, final viscosity; SV, setback viscosity; P_Time_, pasting time; P_Temp_, pasting temperature. Data are given as means of triplicate assays ± SD (standard deviation). Values with different letters in the same column are significantly different (*p* < 0.05). The values are labeled by the letters a to d from the highest to the lowest. The ‘-‘ represent that no value of pasting temperature of the corn powder was displayed in RVA (Rapid Visco Analyser).
